# An integrated analysis of public genomic data unveils a possible functional mechanism of psoriasis risk via a long-range *ERRFI1* enhancer

**DOI:** 10.1186/s12920-020-0662-9

**Published:** 2020-01-22

**Authors:** Naoto Kubota, Mikita Suyama

**Affiliations:** 1 Division of Bioinformatics, Medical Institute of Bioregulation, Kyushu University, Fukuoka, 812-8582 Japan; 2 Research Fellow of Japan Society for the Promotion of Science, Tokyo, 102-0083 Japan

**Keywords:** Psoriasis, GWAS, Public data integration, Functional variant, Regulatory element, *ERRFI1*

## Abstract

**Background:**

Psoriasis is a chronic inflammatory skin disease, for which genome-wide association studies (GWAS) have identified many genetic variants as risk markers. However, the details of underlying molecular mechanisms, especially which variants are functional, are poorly understood.

**Methods:**

We utilized a computational approach to survey psoriasis-associated functional variants that might affect protein functions or gene expression levels. We developed a pipeline by integrating publicly available datasets provided by GWAS Catalog, FANTOM5, GTEx, SNP2TFBS, and DeepBlue. To identify functional variants on exons or splice sites, we used a web-based annotation tool in the Ensembl database. To search for noncoding functional variants within promoters or enhancers, we used eQTL data calculated by GTEx. The data of variants lying on transcription factor binding sites provided by SNP2TFBS were used to predict detailed functions of the variants.

**Results:**

We discovered 22 functional variant candidates, of which 8 were in noncoding regions. We focused on the enhancer variant rs72635708 (T > C) in the 1p36.23 region; this variant is within the enhancer region of the *ERRFI1* gene, which regulates lipid metabolism in the liver and skin morphogenesis via EGF signaling. Further analysis showed that the *ERRFI1* promoter spatially contacts with the enhancer, despite the 170 kb distance between them. We found that this variant lies on the AP-1 complex binding motif and may modulate binding levels.

**Conclusions:**

The minor allele rs72635708 (rs72635708-C) might affect the *ERRFI1* promoter activity, which results in unstable expression of *ERRFI1*, enhancing the risk of psoriasis via disruption of lipid metabolism and skin cell proliferation. Our study represents a successful example of predicting molecular pathogenesis by integration and reanalysis of public data.

## Background

Genome-wide association studies (GWAS) are useful means for identifying trait- or disease-associated single nucleotide polymorphisms (SNPs) [1]. Many GWAS have been conducted for various phenotypes, with a large number of trait-associated SNPs accumulated in public databases, such as the GWAS Catalog [2]. However, understanding why these SNPs are associated with diseases has been difficult because they usually lie within noncoding regions. Such variants are predicted to affect gene expression via *cis*-regulatory elements, such as enhancers or repressors. If the responsible elements and target genes can be identified, noncoding variants may be useful not only as diagnostic markers but also to further shed light on the molecular pathogenesis of diseases. Thus, elucidating the mechanisms of how noncoding variants are associated with diseases is critical.

In recent years, various genomic and epigenetic data have accumulated due to next-generation sequencing (NGS). Epigenetics is the study of heritable changes without changes in the DNA sequence, including DNA methylation, histone modifications, and chromatin remodeling [3]. Advanced epigenetic studies have made it possible to predict the location of *cis-*regulatory elements, such as promoters, enhancers, and repressors in noncoding regions. Furthermore, over the past decade, many experimental methods to discern the higher-order genomic structure have been developed [4]. Hi-C, which is the NGS-based method of capturing genome-wide DNA–DNA interactions, has revealed that the genome is partitioned into megabase-scale structural domains called topologically associating domains (TADs) [5–7]. These data have also been used to detect locality and contact between gene promoters and distant *cis*-regulatory elements in various living tissues and cells. Recent studies have shown that noncoding variants associated with diseases affect the regulation of distant genes, even at megabase distances [8–17]. Therefore, integrated analysis of various epigenetic data, including Hi-C data, can help in the functional annotation of noncoding sequences involved in the long-range regulation of gene expression, disruptions of which lead to various diseases.

In this study, we focused on psoriasis, an autoimmune and chronic inflammatory skin disease characterized by overproliferation of keratinocytes. This is one of the most common diseases in the European population, and the involvement of genetic factors has been strongly suggested, with many susceptibility loci identified by GWAS [18–31]. Several risk markers have been reported to have potential regulatory functions, especially in immune cells [30]. However, the detailed mechanisms governing the onset of psoriasis via *cis*-regulatory elements, in particular which are true causal variants, have not been thoroughly investigated. To understand the molecular pathology of psoriasis and develop therapeutic strategies, we used public data regarding psoriasis risk variants and constructed a computational analysis pipeline to further discover functional variants. We collected variants in high linkage disequilibrium with risk variants and searched for those with changed amino acids, modulated splicing, or variants located in transcriptional regulatory sequences. In particular, for variants of regulatory sequences, we identified target genes using expression quantitative trait loci (eQTL) data and enhancer–promoter correlations. Physical interactions were then validated using Hi-C and ChIA-PET data. Additionally, we searched for transcription factors (TFs) affected by the variants. Through this analysis, we reported several previously unknown functional variants of psoriasis and have shown that long distance transcriptional regulation may be affected by one noncoding variant. This study revealed a new aspect of the molecular pathogenesis of psoriasis.

## Methods

### Psoriasis GWAS SNPs and LD variants

We obtained a summary of tag SNPs associated with psoriasis risk from the GWAS Catalog [2], which is a curated database of published GWAS. The data of variants reported to be associated with “Psoriasis”, “Cutaneous psoriasis”, and “Psoriasis vulgaris” from 2008 to 2018 were used for the following analysis. LDlink [32] was programmatically used to obtain data about variants strongly linked (*r*^2^ > 0.8) with psoriasis risk SNPs in the European population, of which genotype data originated from Phase 3 (Version 5) of the 1000 Genomes Project (Utah Residents from North and West Europe, Toscani in Italia, Finnish in Finland, British in England and Scotland, Iberian population in Spain) [33].

### Exonic and splice site variant annotation

We performed annotation of exonic or splice site variants linked with psoriasis risk SNPs using the Ensembl Variant Effect Predictor [34]. For exonic variants, we extracted deleterious ones, such as missense variants annotated as “probably damaging” or “possibly damaging” per the PolyPhen-2 score [35], frameshift, start lost, and/or stop gained variants. For variants in splice acceptor or donor sites, we defined those, of which differences of the MaxEntScan scores [36] between protective and risk alleles are more than 5, as deleterious splice site variants. The effect on gene expression was assessed using eQTL data provided by GTEx Analysis V7 [37, 38]. The statistical significance was ascertained by GTEx project (https://storage.googleapis.com/gtex-public-data/Portal_Analysis_Methods_v7_09052017.pdf).

### Noncoding variant annotation

For variants in the promoter regions, those located 2 kb upstream of the transcription start site as defined by NCBI RefSeq were used for further analysis. The BED format file of promoter regions was downloaded using the UCSC Table Browser and intersected with the BED format file of variants using BEDTools [39]. For identifying enhancer variants and their targets, we used enhancer–promoter correlation data across a broad panel of cells from the FANTOM5 project [40]. We integrated the promoter or enhancer variant data with eQTL data to obtain variants that affected expression levels of their target genes. Next, we used data regarding variants on TFBSs as analyzed by SNP2TFBS [41] to discover those that affect binding levels of TFs. To verify that the TFBSs were indeed functional, peak files of the corresponding TF ChIP-seq were obtained using DeepBlue API access [42].

### Enrichment analysis

Using ChIP-Atlas [43], we performed the enrichment analysis of histone marks for 104 promoter regions including LD variants and 119 promoter regions associated with enhancers including LD variants. 36,069 promoter regions defined by NCBI RefSeq were used as control. The parameters were set as follows: “Antigen class”- “Histone”, “Cell type Class” – “All cell types”, “Threshold for Significance” – “50”.

### Functional analysis of genomic elements

ChIP-seq signals of six cell lines (GM12878, H1-hESC, HSMM, HUVEC, K562, NHEK, and NHLF) for H3K4me3, H3K4me1, and H3K27ac were displayed by a transparent overlay method. For identifying chromatin states of genomic regions of interest across various tissues and cells, we used the imputed 25-STATE MODEL characterized by ChromHMM software, which can integrate multiple datasets, such as ChIP-seq data of various histone modifications. All tissues or cells we used for the analysis (Liver, Foreskin Fibroblasts, Foreskin Keratinocytes, Foreskin Melanocytes, Adipose Nuclei, Lung, Ovary, Skeletal Muscle Female, Skeletal Muscle Male, Thymus, Pancreatic Islets, Spleen, Stomach Mucosa, Small Intestine, Sigmoid Colon, Hematopoietic stem cells, T helper memory cells from peripheral blood, T CD8+ naive cells from peripheral blood, T cells from cord blood, T cells from peripheral blood, Hematopoietic stem cells G-CSF-mobilized Female, Hematopoietic stem cells G-CSF-mobilized Male, T helper naive cells from peripheral blood, B cells from cord blood, B cells from peripheral blood, Neutrophils from peripheral blood, T helper cells from peripheral blood, Monocytes from peripheral blood, T CD8+ memory cells from peripheral blood) were processed by the Roadmap Epigenomics Project [44]. ChIP-seq data of various proteins (p300, MAFK, MAFF, JUND, MAX, MAZ, MXI1, BHLHE40, CEBPB, COREST, ARID3A, C-JUN, CHD2, BRCA1, SMC3, RFX5, NRF1, Pol2, Pol2 S2, RAD21, TBP, USF2, CEBPZ, and IRF3) for HepG2 cells were visualized using annotation tracks in the UCSC Genome Browser (ENCODE Transcription Factor Binding Tracks, SYDH TFBS). BHLHE40 ChIP-seq signals and peaks for K562, HepG2, GM12878, and A549 cells were also visualized using annotation tracks in the UCSC Genome Browser (ENCODE Transcription Factor Binding Tracks, SYDH TFBS). The logos of the searched motifs, Bach1::Mafk, FOS, JUND, Arnt, and BHLHE40 were generated using WebLogo [45] based on a position frequency matrix provided by the JASPAR CORE database [46] (MA0591.1, MA0476.1, MA0491.1, MA0004.1, and MA0464.2, respectively).

### Quantitation of enhancer and promoter activities

Data of enhancer and promoter activities quantified based on CAGE were obtained using FANTOM Human Promoters (http://slidebase.binf.ku.dk/human_promoters) and FANTOM Human Enhancers (http://slidebase.binf.ku.dk/human_enhancers).

### Visualization of chromatin interactions

Contact profiles of chromosomes in the liver and IMR90 cells were generated from Hi-C data using the 3D Genome Browser [47], and each was visualized with CTCF ChIP-seq signals (ENCODE accession number of Liver CTCF ChIP-seq: ENCFF555SBI and table name of IMR90 CTCF ChIP-seq in the UCSC Genome Browser: wgEncodeSydhTfbsImr90CtcfbIggrabSig). 4C-like outputs of Hi-C data were generated using 3DIV [48]. Pol II ChIA-PET signals and interaction data of K562 cells (wgEncodeGisChiaPetK562Pol2SigRep1, wgEncodeGisChiaPetK562Pol2InteractionsRep1) and MCF-7 cells (wgEncodeGisChiaPetMcf7Pol2SigRep3, wgEncodeGisChiaPetMcf7Pol2InteractionsRep3) were visualized in the UCSC Genome Browser along with the enhancer–promoter correlations data from the FANTOM5 project. The intensity of the lines of ChIA-PET interactions indicated signal strength. CTCF binding motif and their orientations were searched using the JASPAR CORE database.

### Genomic sequence alignments

For comparing multiple species’ genomic sequences, we used the UCSC Genome Browser and GenomeCons [49] to confirm the evolutionary conservation of searched motifs. We used the following species’ genomic sequences (assembly): Human (GRCh37/hg19), Chimp (WUGSC *Pan_troglodytes*-2.1.4/panTro4), Gorilla (gorGor3.1/gorGor3), Orangutan (WUGSC 2.0.2/ponAbe2), Gibbon (GGSC Nleu3.0/nomLeu3), *Rhesus* (BGI CR_1.0/rheMac3), Crab-eating macaque (*Macaca_fascicularis*_5.0/macFas5), Baboon (Baylor Pham_1.0/papHam1), Green monkey (*Chlorocebus_sabeus* 1.0/chlSab1), Mouse (GRCm38/mm10), Rabbit (Broad/oryCun2), Cow (Baylor Btau_4.6.1/bosTau7), Cat (ICGSC *Felis_catus* 6.2/felCat5), Dog (Broad/canFam3), Elephant (Broad/loxAfr3), Chicken (ICGSC *Gallus_gallus*-4.0/galGal4), *X. tropicalis* (JGI 7.0/xenTro7), Zebrafish (Zv9/danRer7), Lamprey (WUGSC 7.0/petMar2).

### Analysis of TF binding levels

We downloaded mapped read data of JUND ChIP-seq (ENCFF263ZVJ) and input (ENCFF235CCD) in bam format from the ENCODE database and used MACS2 [50] for peak calling (*Q* < 0.01). The called peaks that contained “TGAGTCAT” or “TGAGTCAC” sequences were used to compare signal strengths. Peaks containing both motifs were excluded from the analysis. We used the GGGenome (https://gggenome.dbcls.jp/ja/) to obtain BED format files of the sequences of interest, and BEDTools was used to extract peaks containing the motifs. We used the Mann–Whitney U test to compare their signal strengths (*P* < 0.05).

### Statistical tests, visualization, and tools used

Development of an analysis pipeline and all statistical tests were done in the Python 3.7 and the GNU Bash 3.2 environment. All graphs except for the GTEx violin plots were made using the seaborn Python package.

## Results

### A computational genome-wide survey of functional variants by integrating multiple public datasets

**Fig. 1:**
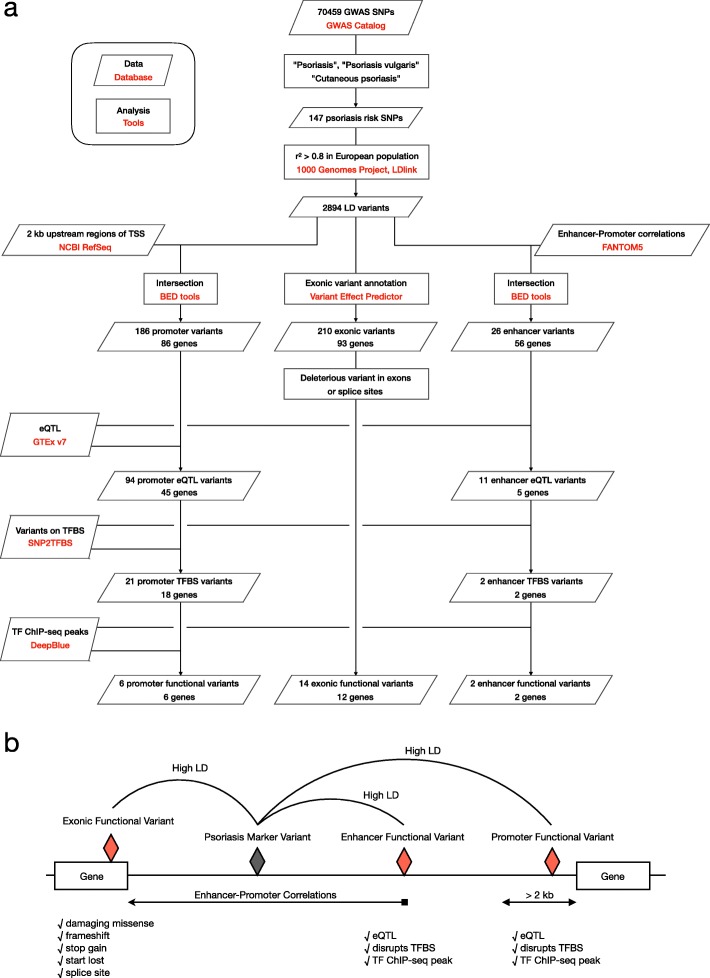
An overview of this study. **a** Schematic diagram of the analysis pipeline integrating multiple public datasets. **b** Functional variants to be searched in this analysis pipeline. For gene regions, we searched for deleterious missense, frameshift, stop gain, start lost, and splice site variants. For promoters and enhancers, we looked for variants called eQTLs and those that alter the binding sequences of TFs. The binding of the TFs were confirmed using public ChIP-seq peaks data

Using a variety of public genomic datasets, we constructed a pipeline to comprehensively identify functional variants (Fig. 1a). First, SNPs reported to be associated with psoriasis were extracted from GWAS Catalog-registered data, and then variants with high linkage disequilibrium were obtained and used in subsequent analysis. As for the gene regions, we surveyed genetic variants that lead to amino acid substitutions expected to affect protein structure and function; in addition, variants at splice sites were also explored. As for regulatory variants, we looked for those located in active enhancers that disrupted binding motifs for TFs and were expected to affect binding affinities. Although few previous studies have been conducted, the bindings of TFs were confirmed by comprehensively obtaining peak files of corresponding ChIP-seq data using the DeepBlue web server [42]. Through this analysis, we attempted to identify functional variants, TFs, and verified target genes (Fig. 1b).

### Candidate functional variants newly identified in gene regions

We identified 14 deleterious variants in the gene regions (Table 1) and some of them affected their own gene expression (Additional file 1: Figure S1). Most variants were previously reported to be associated with autoimmune diseases, but some were newly identified. An identified variant, rs2549797, was highly linked with psoriasis risk SNP rs2910686 [25] (*r*^2^ = 0.8051) and located near the 5′ canonical splice site of *ERAP2* gene exon 15, forming a new splice site (Additional file 1: Figure S2a). This variant remarkably altered the signal strength of the splice site from − 1.440 (for risk allele A) to 5.463 (for protective allele G) (Additional file 1: Figure S2b). The transcript derived from the risk allele (rs2549797-A) encodes for the full-length ERAP2 protein. The transcript derived from the protective allele (rs2549797-G) contains a premature stop codon that might cause nonsense-mediated decay (NMD) [51]. In addition, we found that rs2549797 is completely linked with rs2248374 (*r*^2^ = 1.0000) located on the 5′ canonical splice site of *ERAP2* gene exon 10, which was previously reported to alter the signal strength of the canonical splice site and a noncanonical transcript containing a premature stop codon, leading to NMD [52]. Referring to the GTEx eQTL data, these completely linked variants that might cause NMD (rs2549797-G and rs2248374-G) markedly decreased *ERAP2* gene expression in all analyzed tissues and cells (Additional file 1: Figure S1). *ERAP2* encodes for endoplasmic reticulum aminopeptidase 2, which is responsible for trimming peptides to optimal sizes for antigen presentation by MHC class I [53]. *ERAP2* variants are associated with various immune diseases, including psoriasis [54, 55]. Together, these results showed that the protective haplotype (rs2549797-G and rs2248374-G) almost certainly caused NMD to decrease *ERAP2* gene expression, which appeared to reduce the risk of psoriasis.

**Table 1:** Candidate functional variants in exons or splice sites associated with psoriasis risk

Chr.	Position	Variant	Alleles (P/R)	RAF	Gene	AA	InterPro annotation	Marker (*P*-value, Study)	r^2^
Known functional variants									
Splice site									
6	31,380,000	rs199503730	G/−	0.0447	MICA	5′ ss	–	rs2395029 (2.00E-26, [19])	0.8860
Exon									
1	25,291,010	rs6672420	A/T	0.4791	RUNX3	I/N	–	rs7536201 (2.00E-12, [25]; 2.00E-08, [29])	0.9046
1	67,705,958	rs11209026	A/G	0.9384	IL23R	Q/R	–	rs11209026 (7.00E-07, [24])	1.0000
								rs9988642 (1.00E-26 [25]; 7.00E-14, [29])	0.8520
6	31,473,957	rs3134900	(C/G)?	NR	MICB	I/M	MHC class I-like antigen recognition-like, MHC classes I/II-like antigen recognition protein	rs3134792 (1.00E-09, [18])	0.8008
6	111,913,262	rs33980500	C/T	0.0855	TRAF3IP2	D/N	–	rs33980500 (1.00E-16, [23]; 4.00E-45, [25]; 1.00E-23, [29])	1.0000
17	78,178,893	rs11652075	T/C	0.5099	CARD14	W/R	–	rs11652075 (3.00E-08, [25])	1.0000
19	10,463,118	rs34536443	C/G	0.9712	TYK2	A/P	Protein kinase domain, Protein kinase-like domain, Serine–threonine/tyrosine–protein kinase, catalytic domain, Tyrosine–protein kinase	rs34536443 (9.00E-31, [25])	1.0000
19	10,469,975	rs12720356	C/A	0.9076	TYK2	S/I	Protein kinase domain, Protein kinase-like domain, Serine–threonine/tyrosine–protein kinase, catalytic domain, Tyrosine–protein kinase	rs12720356 (4.00E-11, [25])	1.0000
19	49,206,674	rs601338	G/A	0.4414	FUT2	W/*	–	rs492602 (7.00E-13, [30])	0.9920
19	49,206,985	rs602662	G/A	0.4682	FUT2	G/S	–	rs492602 (7.00E-13, [30])	0.8822
22	21,982,892	rs2298428	C/T	0.1769	YDJC	A/T	Glycoside hydrolase/deacetylase, beta/alpha-barrel	rs181359 (2.00E-07, [29])	0.8941
								rs4821124 (4.00E-08, [25])	0.9477
Newly identified functional variants									
Splice site									
5	96,245,518	rs2549797	G/A	0.5199	ERAP2	5′ ss	–	rs2910686 (2.00E-08, [25])	0.8051
Exon									
12	5,660,905	rs60542959	T/G	0.9344	COQ10A	I/M	–	rs2066807 (5.00E-12, [27])	0.9523
								rs2066808 (6.00E-10, [26])	0.9388
								rs2066819 (5.00E-17, [25])	0.9517
17	73,874,071	rs4600514	G/A	0.1650	TRIM47	R/W	B-box-type zinc finger, Zinc finger RING/FYVE/PHD-type	rs55823223 (1.00E-08, [30])	0.9075

An asterisk in “AA” column indicates a stop codon. As for rs3134792 and its functional variants, the direction of effects is unknown

*P/R* protective/risk, *RAF* risk allele frequency, *NR* not reported, *AA* amino acids, *5′ ss* 5 prime splice site, *r*^*2*^ r-squared value between functional variants and GWAS variants

We also identified the rs60542959 variant in the *COQ10A* gene region. The protective allele rs60542959-T changes the first codon ATG (methionine) to ATT (isoleucine), causing a start lost mutation. This variant is highly linked with three psoriasis risk SNPs (rs2066807, rs2066808, and rs2066819; *r*^2^ = 0.9523, 0.9388, and 0.9517, respectively) [21, 24–27, 29] located within the *STAT2* gene region (Additional file 1: Figure S3a). The *COQ10A* gene has two transcript variants (NM_144576.3, NM_001099337.1) (Additional file 1: Figure S3b), and the transcript NM_144576.3 that has a start lost mutation by rs60542959 is highly transcribed in natural killer cells, T cells, and lymphocytes of B cell lineage, whereas another transcript NM_001099337.1 is not (Additional file 1: Figure S3c–d). This suggested that rs60542959 may have a significant effect on *COQ10A* expression in immune cells. The start codon is widely conserved among mammals, indicating the functional importance of this transcript (Additional file 1: Figure S3e). The GTEx eQTL data showed that the rs60542959-T allele significantly reduces *COQ10A* gene expression in one tissue type (esophagus mucosa) (Additional file 1: Figure S1, S3f). *COQ10A* encodes for coenzyme Q-binding protein homolog A, which is required for coenzyme Q function in the respiratory chain [56]. Several associations of coenzyme Q10 with immune function have been reported [57, 58]. The association between *COQ10A* and psoriasis is unclear, but it may have some impact on risk.

### Candidate functional variants in promoters and enhancers

For regulatory sequence variants, we looked for those located within promoters or enhancers and changed the expression level of target genes. Previous studies reported that GWAS-identified noncoding SNPs are enriched among enhancers in several types of immune cells (e.g., CD8+ T cells and CD4+ T cells) [30]. We also performed the enrichment analysis using ChIP-Atlas function [43]. The results showed that H3K27ac ChIP-seq peaks were enriched among promoter regions including LD variants and promoter regions associated with enhancers including LD variants in several types of blood cells, including T cells (Additional file 1: Figure S4). This indicated that our pipeline reproduced previous findings for noncoding variants on enhancers in immune cells.

We identified 6 variants in promoters and the associated TFs whose binding sites were disrupted by these variants (Table 2, Additional file 1: Figure S5–S6). We found that rs3132089, a promoter variant of the *HCP5* gene, is highly linked with psoriasis risk SNP rs3134792 (*r*^2^ = 0.9253) [18] (Additional file 1: Figure S7a) and located on the binding motif of ARNT and BHLHE40 proteins, which is predicted to result in decreased binding (Additional file 1: Figure S7b). ChIP-seq data showed that BHLHE40 binds to the motif in K562 and HepG2 cells (Additional file 1: Figure S6, S7c). Its minor allele (rs3132089-A) decreases *HCP5* expression in the thyroid and sun exposed skin of lower leg (Additional file 1: Figure S5, S7d). *HCP5* encodes for a long noncoding RNA, which is highly expressed in immune cells (Additional file 1: Figure S7e) and involved in inflammatory, adaptive, and innate immune responses [59, 60]. A variant rs2395029, located on *HCP5* gene body, were previously reported to be associated with psoriasis [19]. Although the molecular mechanism of how *HCP5* transcript controls immune responses remains unclear, these findings provided evidence that the differential expression of *HCP5* depending on rs3132089 genotype in the skin alters the immune response level, leading to the increased/decreased risk of developing psoriasis.

**Table 2:** Candidate functional variants in promoters associated with psoriasis risk

Chr.	Position	Variant	Alleles (P/R)	RAF	Gene	TF	Marker (*P*-value, Study)	r^2^
6	31,371,100	rs6906175	G/C	0.2952	MICA	REST	rs13437088 (3.00E-40, [25])	1.0000
6	31,430,009	rs3132089	(G/A)?	NR	HCP5	ARNT, BHLHE40	rs3134792 (1.00E-09, [18])	0.9253
6	31,462,134	rs3130923	(G/A)?	NR	MICB	TCF3	rs3134792 (1.00E-09, [18])	0.8762
12	56,728,257	rs11358218	A/−	0.9304	PAN2	SPI1	rs2066807 (5.00E-12, [27])	0.9541
							rs2066808 (6.00E-10, [26])	1.0000
							rs2066819 (5.00E-17, [25])	0.8933
16	30,968,588	rs4889599	C/T	0.3658	SETD1A	EGR1	rs10782001 (9.00E-10, [22])	0.9703
							rs12445568 (1.00E-16, [25])	0.8876
							rs13708 (2.00E-08, [27])	0.9915
20	43,989,513	rs2741432	G/A	0.7744	SYS1	CEBPB	rs1008953 (1.00E-07, [22])	0.9943

As for rs3134792 and its functional variants, the direction of effects is unknown

*P/R* protective/risk, *RAF* risk allele frequency, *NR* not reported, *TF* transcription factor, *r*^*2*^ r-squared value between functional variants and GWAS variants

**Fig. 2:**
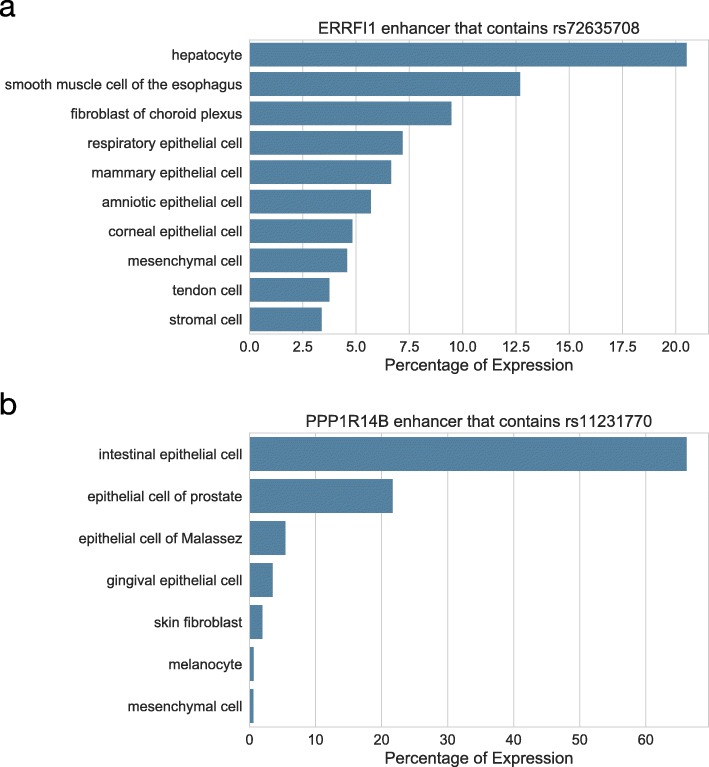
The eRNA-based quantified activity of enhancers. The “Percentage of Expression” for each cell type refers to how much of the total expression (normalized CAGE counts from all cells) the enhancer emits for each cell type. **a** The top 10 cells highly expressing eRNA from a CAGE-defined enhancer (hg19: chr1: 8,257,762-8,258,040) in which rs72635708 is located. **b** The top 7 cells highly expressing eRNA from a CAGE-defined enhancer (hg19: chr11: 64,140,304-64,140,795) in which rs11231770 is located

We identified two candidate functional variants in enhancers and associated TFs whose binding sites were disrupted by the variants (Table 3). To identify target genes of enhancers that contain functional variants of psoriasis risk, we analyzed enhancer–promoter correlations using CAGE datasets provided by the FANTOM5 project [40]. In the FANTOM5 project, CAGE technology was used for quantitative detection of transcribed enhancers (eRNA) in high nucleotide resolution. The CAGE-defined transcripts dataset enables direct pairwise correlation between enhancers and putative target genes. The identified enhancer variants were rs72635708 and rs11231770, which target the *ERRFI1* and *PPP1R14B* genes, respectively. Based on eRNA expression levels, we found that the enhancer element in which rs72635708 is located was highly active in the hepatocytes, fibroblasts, and various epithelial cells, and the enhancer element in which rs11231770 is located was highly active in various epithelial cells, especially in the intestinal epithelial cells (Fig. 2). Although the identified variants might be involved in psoriasis risk, we focused on rs72635708 and the *ERRFI1* gene for further analyses for ease of interpretation of the association with psoriasis risk.

**Table 3:** Candidate functional variants in enhancers associated with psoriasis risk

Chr.	Position	Variant	Alleles (P/R)	RAF	Gene	TF	Marker (*P*-value, Study)	r^2^
1	8,257,959	rs72635708	T/C	0.2286	ERRFI1	ARNT, BACH1, FOS, MAFK, NFE2	rs417065 (8.00E-07, [27])	0.9832
11	64,140,624	rs11231770	A/G	0.6292	PPP1R14B	SP1, SP2, ZNF263	rs645078 (2.00E-06, [25])	1.0000

*P/R* protective/risk, *RAF* risk allele frequency, *NR* not reported, *TF* transcription factor, *r*^*2*^ r-squared value between functional variants and GWAS variants

### A candidate functional variant rs72635708 and the *ERRFI1* gene are within the same spatial regulatory unit

**Fig. 3:**
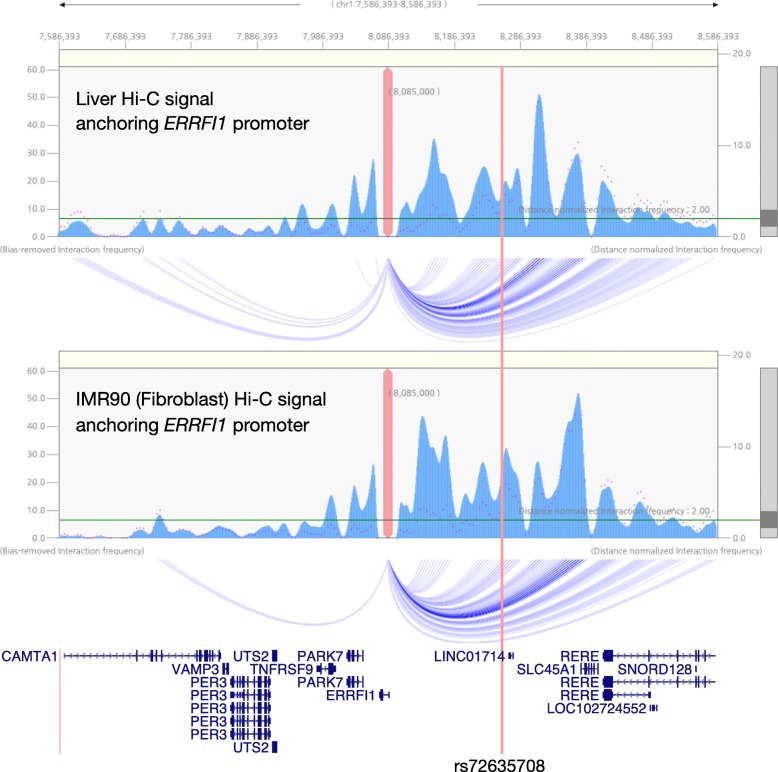
The enhancer, in which candidate functional variant rs72635708 is located, physically interacts with the *ERRFI1* promoter in the liver and fibroblasts. The upper panel and middle panel are 4C-like representations of Hi-C data for the liver and IMR90 cell line by anchoring the *ERRFI1* promoter. The blue waveform represents the bias-removed interaction frequency, and the magenta dot represents the distance normalized interaction frequency. Regions where the distance normalized interaction frequency exceeds two are connected to the *ERRFI1* promoter by blue arcs. The bottom panel is the RefSeq Curated Genes annotation track. The location of candidate functional variant rs72635708 is indicated by a vertical red line

A candidate variant rs72635708, which is in the 1p36.23 region, is located within an enhancer element targeting the *ERRFI1* gene, despite the 170 kb distance between them. This variant is highly linked with two risk SNPs (rs417065 and rs11121129; *r*^2^ = 0.9832 and 0.6547, respectively) identified by two independent GWAS [25, 27], strongly supporting that this is a susceptibility locus of psoriasis. To further confirm the functional interaction between the enhancer element and the *ERRFI1* promoter, we analyzed Hi-C and ChIA-PET data to detect the spatial structure of chromosomes. The higher-order structure of chromosomes and gene function are closely related; thus, it is very important to use chromosome conformation data to confirm physical contact in order to detect enhancer–promoter interactions. We tested spatial contacts between this enhancer and the *ERRFI1* promoter using Hi-C data for the liver and IMR90, which is an immortalized cell line derived from fibroblasts. The Hi-C contact map showed that the *ERRFI1* promoter and a candidate functional variant rs72635708 are located on the same TAD, which is a spatial functional unit of chromosomes [6, 7, 61], in both the liver and IMR90 (Additional file 1: Figure S8). The prominent peaks of CTCF ChIP-seq signals—CTCF is well known as an insulator protein that defines TADs, and its polarity determines the genome looping [62]—were found at distinct boundary regions of the TAD structure. 4C-like representation of Hi-C data by anchoring the *ERRFI1* promoter also supported the long-range contact between the candidate functional variant and *ERRFI1* promoter in the liver and IMR90 (Fig. 3). Focusing on RNA polymerase II ChIA-PET signals in this region, there was no enhancer–promoter contact in K562, an immortalized cell line derived from blood cells, but there was a strong contact signal in MCF-7, an immortalized cell line derived from mammary epithelial cells (Additional file 1: Figure S9). Combined with enhancer–promoter correlation data defined by eRNA and mRNA expression profiles, we observed that there was a strong functional interaction between the *ERRFI1* promoter and the enhancer that contains rs72635708. In addition to these results, binding regions and motif orientations of CTCF were also explored, and we found distinct boundary regions of TAD structure with convergent orientations of the CTCF binding motifs (Additional file 1: Figure S9). Taken together, these results strongly suggested that the enhancer in which the predicted functional variant rs72635708 is located belongs to the same regulatory unit as the *ERRFI1* gene and may directly regulate its expression level, despite the long distance between them, especially in the liver, fibroblasts, and epithelial cells, but not in blood cells.

### rs72635708 may affect *ERRFI1* expression by modulating the binding level of the AP-1 complex

**Fig. 4:**
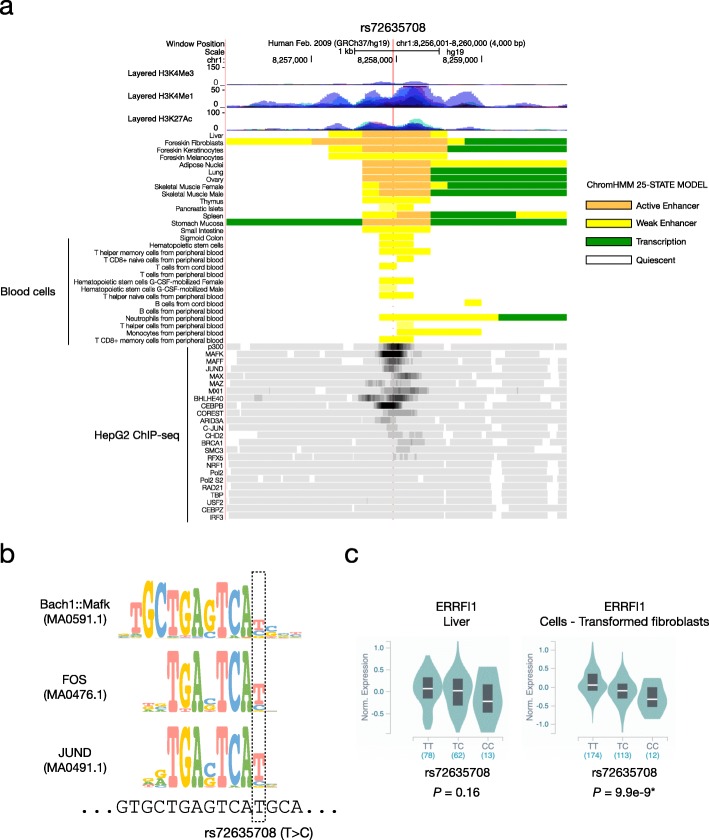
The functional impact of rs72635708. **a** At the top, the layered ChIP-seq signals of H3K4me3, H3K4me1, and H3K27ac are shown. At the middle, ChromHMM annotation (25-STATE MODEL) for various tissues and cells is shown. At the bottom, ChIP-seq density signals of various proteins for the HepG2 cell line are shown. The location of rs72635708 is indicated by a vertical red line. **b** The sequence logos based on the position weight matrix of binding sites for Bach1::Mafk (MA0591.1), FOS (MA0476.1), and JUND (MA0491.1) are represented with genomic sequences. The location of rs72635708 is indicated by a dotted box. **c** Comparison of *ERRFI1* expression levels among rs72635708 genotypes in the liver and fibroblasts. The plots were generated by the GTEx project. The horizontal axis shows genotypes with the number of individuals in parentheses. The asterisk indicates statistical significance

Next, we examined the genomic and epigenetic features of the enhancer element in which rs72635708 is located in order to understand its effects on the enhancer. We used chromatin state data as defined by ChromHMM [63], and high enhancer activity was found in many tissues, including the liver and skin fibroblasts, but not in various blood cells (Fig. 4a). ChIP-seq data from HepG2, an immortalized cell line derived from hepatocytes, showed binding of many proteins, including p300, which is an active element marker that functions as histone acetyltransferase, and MAFK, MAFF, and JUND, which are members of the AP-1 complex family. As shown in Table 3, when combined with TFBS logos based on the position frequency matrix, we found that the rs72635708 risk allele (rs72635708-C) disrupts the binding motif of the AP-1 complex (Fig. 4b). This binding motif was strongly conserved among primates, indicating its important role retained through evolution (Additional file 1: Figure S10a). To investigate the effect of the risk allele rs72635708-C on enhancer activity, we analyzed the binding level of JUND by using ChIP-seq peak data. We selected MACS2 peaks containing “TGAGTCAT” (protective form) or “TGAGTCAC” (risk form) from all genomic regions and compared the signal strength between the motifs (excluded peaks containing both motifs). The result showed that the signal strength of peaks with motifs in the risk form was significantly lower than that with motifs in the protective form (*P* < 0.001, Mann–Whitney U test) (Additional file 1: Figure S10b), suggesting that at this locus, JUND binding to the enhancer element might be affected by the rs72635708 risk allele. Furthermore, referring to the results of previously reported allele-specific mapping of DNase-seq [64], this variant was strongly predicted to affect TFs occupancy. This result was consistent with previous reports stating that the AP-1 complex recruits chromatin remodeling factors and alters chromatin accessibility of regulatory sequences [65, 66]. Taken together, we suggested that rs72635708-C may decrease enhancer activity by altering the binding affinity of the AP-1 complex and reducing chromatin accessibility. Regarding the effect of rs72635708 on *ERRFI1* expression, no significant difference but a decreasing trend was observed in the liver, and a significant effect was observed in fibroblasts (Fig. 4c).

## Discussion

Many trait-associated SNPs have been identified by GWAS. However, the roles of these SNPs in molecular pathogenesis have not been clearly interpreted because they lie within noncoding sequences. Herein, we constructed a pipeline integrating different types of public datasets to survey functional variants from GWAS SNPs. As a result, we identified several functional variants involved in psoriasis risk present in both gene and regulatory regions. Although GWAS Catalog contains many suggestive SNPs that do not satisfy genome-wide significant threshold, we confirmed that all psoriasis risk markers linked with identified functional variants have strong signals with significant *p*-values in each GWAS (Tables 1, 2 and 3). These results are predictions, so the validation using independent cohorts or functional experiments will be needed to make a new aspect of the molecular pathogenesis of psoriasis more convincing.

**Fig. 5:**
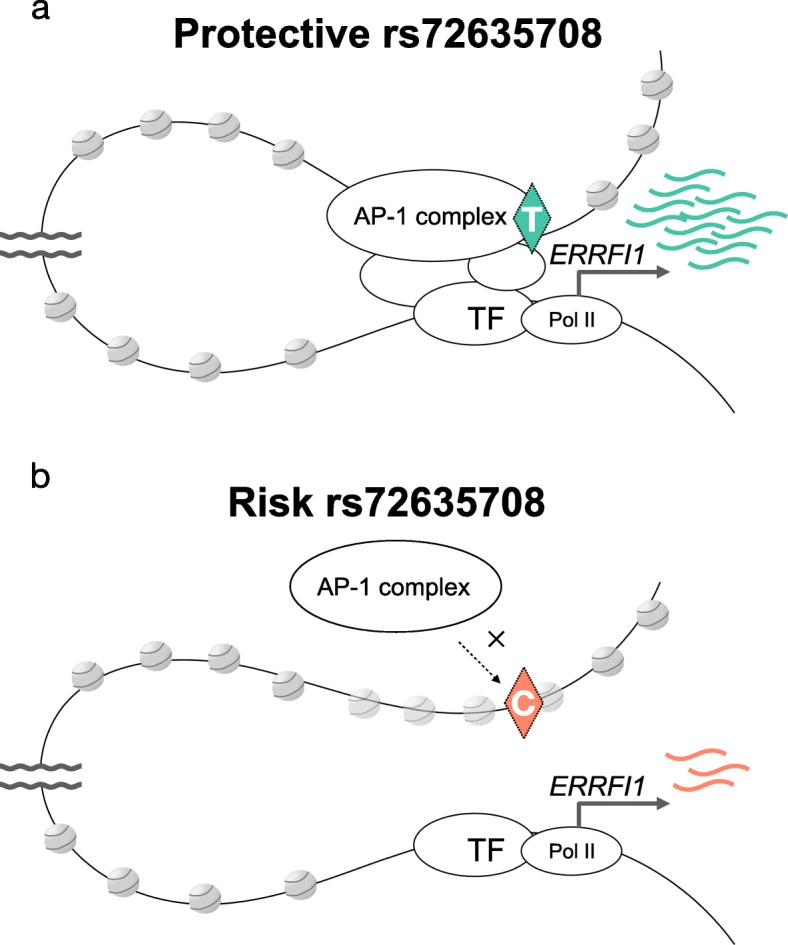
A model for the regulation of *ERRFI1* promoter activity by a long-range enhancer. Chromatin illustrations were obtained from the TogoPictureGallery (https://togotv.dbcls.jp/togopic.2017.10.html). **a** With a protective allele (rs72635708-T), the AP-1 complex can bind to the enhancer element. This leads to chromatin remodeling around the element, resulting in the stable expression of *ERRFI1*. **b** With a risk allele (rs72635708-C), the binding levels of the AP-1 complex to the enhancer element are reduced so that chromatin accessibility might not be enough to be activated by other TFs. This results in the unstable expression of *ERRFI1*

In particular, we proposed a model for the genetic mechanism of psoriasis risk SNP rs417065 and functional variant rs72635708 via a long-range *ERRFI1* enhancer in the 1p36.23 region. In cells with a protective allele (rs72635708-T), the AP-1 complex binds to the enhancer element and leads to chromatin remodeling and other protein binding, resulting in stable expression of the *ERRFI1* gene (Fig. 5a). However, in cells with a risk allele (rs72635708-C), the AP-1 complex may not access the enhancer element, and chromatin accessibility might not be enough to be activated by other proteins, which causes unstable expression of the *ERRFI1* gene (Fig. 5b). Thus, we concluded that *ERRFI1* is a novel target gene for psoriasis risk SNPs and involved in the pathogenesis in hepatocytes and fibroblasts. Many studies have focused on immune cells since psoriasis is an autoimmune disease, but our analysis highlighted the importance of functional variants in other cells, such as hepatocytes and fibroblasts. For further support for our hypothesis, the AP-1 complex ChIA-PET data gleaned from hepatocytes and fibroblasts would be required for validating the predicted interaction between the enhancer and *ERRFI1* promoter.

*ERRFI1*, also known as *Mig-6*, is a ubiquitously expressed gene and well known as an EGF and Erbb signaling inhibitor that binds directly to EGFR and HER2 [67–69]. A previous study reported that *Errfi1* gene knockout in mice caused the hyperactivation of EGF signaling, leading to overproliferation and impaired differentiation of skin cells [70]. In human tissues, *ERRFI1* expression is downregulated in various cancers and in skin with chronic inflammation [70], supporting a role for negative regulation of cell proliferation. Thus, we speculated that the functional variant rs72635708 might target *ERRFI1*. Moreover, it has been reported that *ERRFI1* regulates cholesterol and glucose metabolism in the liver. *Errfi1* liver-specific knockout in mice altered expression of lipid and glucose metabolism-related genes, resulting in increased blood cholesterol and fasting glucose levels and leading to insulin resistance [71–73]. In recent years, psoriasis has been strongly suggested for clinical association with nonalcoholic fatty liver disease (NAFLD) [74], type 2 diabetes (T2D) [75, 76], and metabolic syndrome [77]. Thus, it is assumed that decreased *ERRFI1* expression levels, attributed to the distant enhancer containing the risk variant (rs72635708-C) that reduces transcriptional activity, might lead to the acquisition of insulin resistance and overproliferation of epithelial cells, recapitulating a condition that is clinically considered to induce a high risk for psoriasis.

Additionally, *ERRFI1* is deeply involved in various physiological and pathogenic events, such as lung development [78], endometrial epithelial cell proliferation [79], lung tumorigenesis [80–82], gliomagenesis [83, 84], hepatocellular carcinoma [85, 86], and neointimal hyperplasia in vascular smooth muscle cells [87]. Unveiling the regulatory landscape of *ERRFI1* could contribute to further understanding of the molecular basis of these events. Notably, it has been reported that upregulation of *ERRFI1* is associated with acquired resistance to the EGFR tyrosine kinase inhibitor (TKI) in a cancer cell line [88]. Therefore, rs726365708 might be a useful genetic marker to detect *ERRFI1* activity and the level of EGFR-TKI resistance, enabling personalized clinical application of EGFR-TKIs for patients with cancer or psoriasis.

Although HLA gene variants have been reported as a risk factor for psoriasis in previous studies [89], we were unable to identify exonic functional variants of HLA genes in our analysis pipeline. Since the LDlink, which is a software used in the present analysis, could detect only linkage disequilibrium of biallelic variants, it seemed that the variants of HLA genes, which usually have more than 2 alleles, could not be detected. By developing new software for calculating linkage disequilibrium for variants with more than 2 alleles, we will be able to search for functional variants that we missed in this study. In addition, some of the functional variants identified in the present analysis were concentrated in the nearby chromosomal regions. For example, we identified 1 splice site variants, 2 exonic variants, and 3 promoter variants on chromosome 6, and whether they are independent is not clear. Thus, in future, prioritization procedure will be needed in our pipeline for identifying true causal variants.

## Conclusions

In this study, we investigated the genetic mechanism of psoriasis, especially detailing how noncoding SNPs influence enhancer activity and target genes via a computational approach. We would like to emphasize that it is important to integrate and reanalyze public data to study genetic diseases. This study showed that such analyses can reveal molecular pathogenesis. Although this study focused on one disease, our approach can be applied to other disease-associated SNPs and phenotypes of which pathogenesis remains unclear.

## Supplementary information

**Additional file 1: Figure S1** A heat map of the normalized effect size of GTEx eQTLs for functional variants in exons and splice sites. **Figure S2** rs2549797 is a candidate functional variant located on a splice site of the *ERAP2* gene. **Figure S3** rs60542959 is a deleterious variant that causes a start lost mutation in the *COQ10A* gene. **Figure S4** H3K27ac ChIP-seq peaks of immune cells are enriched among psoriasis-associated promoter regions. **Figure S5** A heat map of the normalized effect size of GTEx eQTLs for functional variants in promoters and enhancers. **Figure S6** A Table of bindings of transcription factors (TFs) to functional variants in promoters and enhancers in various cells. **Figure S7** The rs3132089 is a candidate functional variant located in the *HCP5* gene promoter. **Figure S8** Hi-C contact maps for the liver and IMR90 cell line in the 1p36 region. **Figure S9** An overview of the 1p36.23 region that contains the *ERRFI1* gene and rs72635708. **Figure S10** The effect of rs72635708 on AP-1 complex binding.

## Data Availability

All data used in this study are publically available. A summary file of GWAS Catalog is available at https://www.ebi.ac.uk/gwas/docs/file-downloads and the file name used in this study is “gwas_catalog_v1.0.2-associations_e93_r2019-01-11.tsv”. GTEx single-tissue *cis*-eQTL data is available at https://storage.googleapis.com/gtex_analysis_v7/single_tissue_eqtl_data/GTEx_Analysis_v7_eQTL.tar.gz and the dbGaP accession number is “phs000424.v7.p2”. Enhancer–promoter correlation data across a broad panel of cells from the FANTOM5 project is available at http://slidebase.binf.ku.dk/human_enhancers/presets/serve/hg19_enhancer_promoter_correlations_distances_cell_type.txt.gz . All ChIP-seq data used in this study is available at https://www.encodeproject.org/ and http://www.roadmapepigenomics.org/ . The data in DeepBlue is available at https://deepblue.mpi-inf.mpg.de/dashboard.php#ajax/dashboard.php.

## Abbreviations

eQTL: Expression quantitative trait locus
eRNA: Enhancer RNA
GWAS: Genome-wide association study
LD: Linkage disequilibrium
NGS: Next-generation sequencing
NMD: Nonsense-mediated decay
SNP: Single nucleotide polymorphism
TAD: Topologically associating domain
TF: Transcription factor
TFBS: Transcription factor binding site
