# Supplementary material for: An integrated analysis of public genomic data unveils a possible functional mechanism of psoriasis risk via a long-range ERRFI1 enhancer
Source: BMC Med Genomics. 2020 Jan 22;13:8. doi: 10.1186/s12920-020-0662-9 (PMC6977261; doi:10.1186/s12920-020-0662-9)
Supplement: Supplementary file 1 — Additional file 1: Figure S1 A heat map of the normalized effect size of GTEx eQTLs for functional variants in exons and splice sites. Figure S2 rs2549797 is a candidate functional variant located on a splice site of the ERAP2 gene. Figure S3 rs60542959 is a deleterious variant that causes a start lost mutation in the COQ10A gene. Figure S4 H3K27ac ChIP-seq peaks of immune cells are enriched among psoriasis-associated promoter regions. Figure S5 A heat map of the normalized effect size of GTEx eQTLs for functional variants in promoters and enhancers. Figure S6 A Table of bindings of transcription factors (TFs) to functional variants in promoters and enhancers in various cells. Figure S7 The rs3132089 is a candidate functional variant located in the HCP5 gene promoter. Figure S8 Hi-C contact maps for the liver and IMR90 cell line in the 1p36 region. Figure S9 An overview of the 1p36.23 region that contains the ERRFI1 gene and rs72635708. Figure S10 The effect of rs72635708 on AP-1 complex binding. [file 12920_2020_662_MOESM1_ESM.pdf]

# Supplementary information

An integrated analysis of public genomic data unveils a possible functional mechanism of psoriasis risk *via* a long-range *ERRFI1* enhancer

Supplementary figure (1~10)

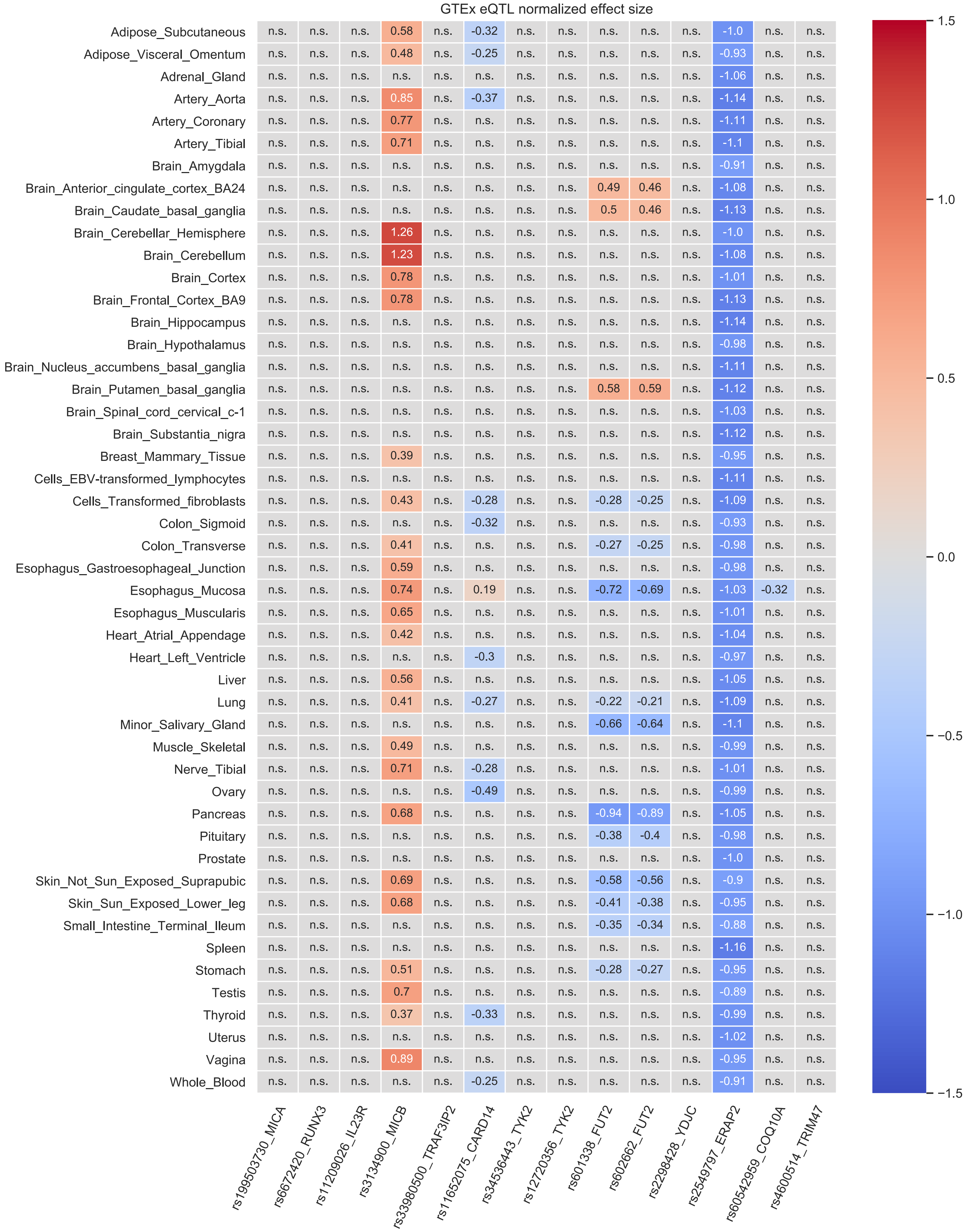


**Fig. 1** A heat map of the normalized effect size of GTEx eQTLs for functional variants in exons and splice sites. The vertical axis represents the tissues analyzed, and the horizontal axis represents the functional variants and their target genes. The score for each cell indicates the normalized effect size, which is the effect of the alternative allele for gene expression. Increases in gene expression are shown in red and decreases in gene expression are shown in blue. The “n.s.” in gray cells mean that the effect of allele on gene expression is not statistically significant.

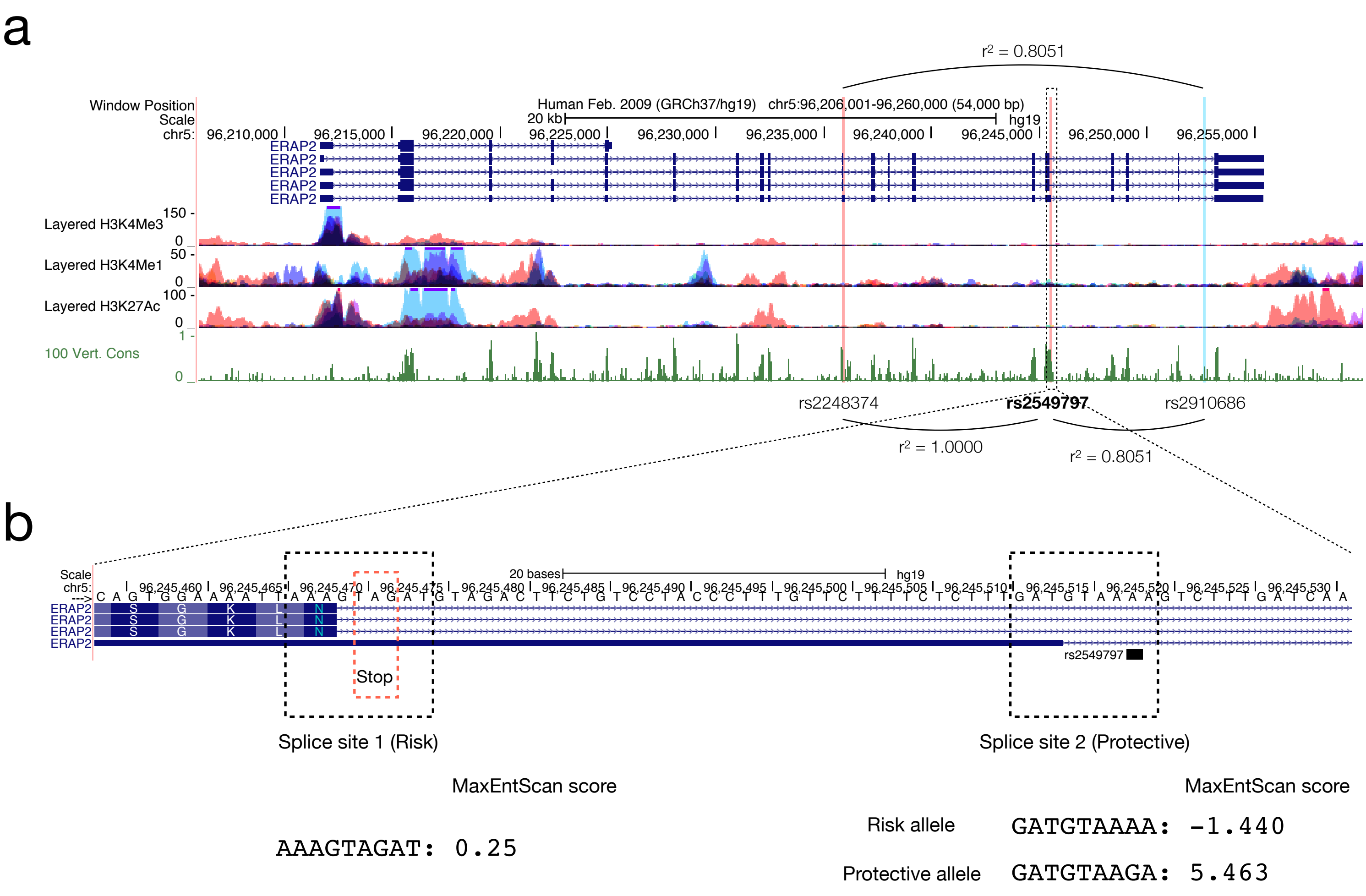


**Fig. 2** rs2549797 is a candidate functional variant located on a splice site of the *ERAP2* gene.

**a.** An overview of the *ERAP2* gene locus with a genome conservation track (100 vertebrates conservation by PhastCons) and layered ChIP-seq signals of H3K4me3, H3K4me1, and H3K27ac in the UCSC Genome Browser. The vertical light blue line indicates the location of the psoriasis GWAS variant rs2910686, and the vertical red lines indicate the location of candidate functional variants. rs2248374 is a splice site variant previously reported to cause nonsense-mediated decay (NMD) [Andrés *et al.*, 2010]. The r-squared values between each variant in the European population, of which genotype data originate from Phase 3 (Version 5) of the 1000 Genomes Project, are also shown.

**b.** A magnified view of the 5′ splice site of exon 15 of the *ERAP2* gene. The black dotted boxes indicate splice site 1 (risk) and splice site 2 (protective). The MaxEntScan score is displayed for each splice site. The score of a sequence that contains the rs2549797 protective allele (rs2549797-G) is larger than splice site 1 (5.463 > 0.25) and thus expected to result in splicing at splice site 2, form a premature stop codon (red dotted box), and cause NMD.

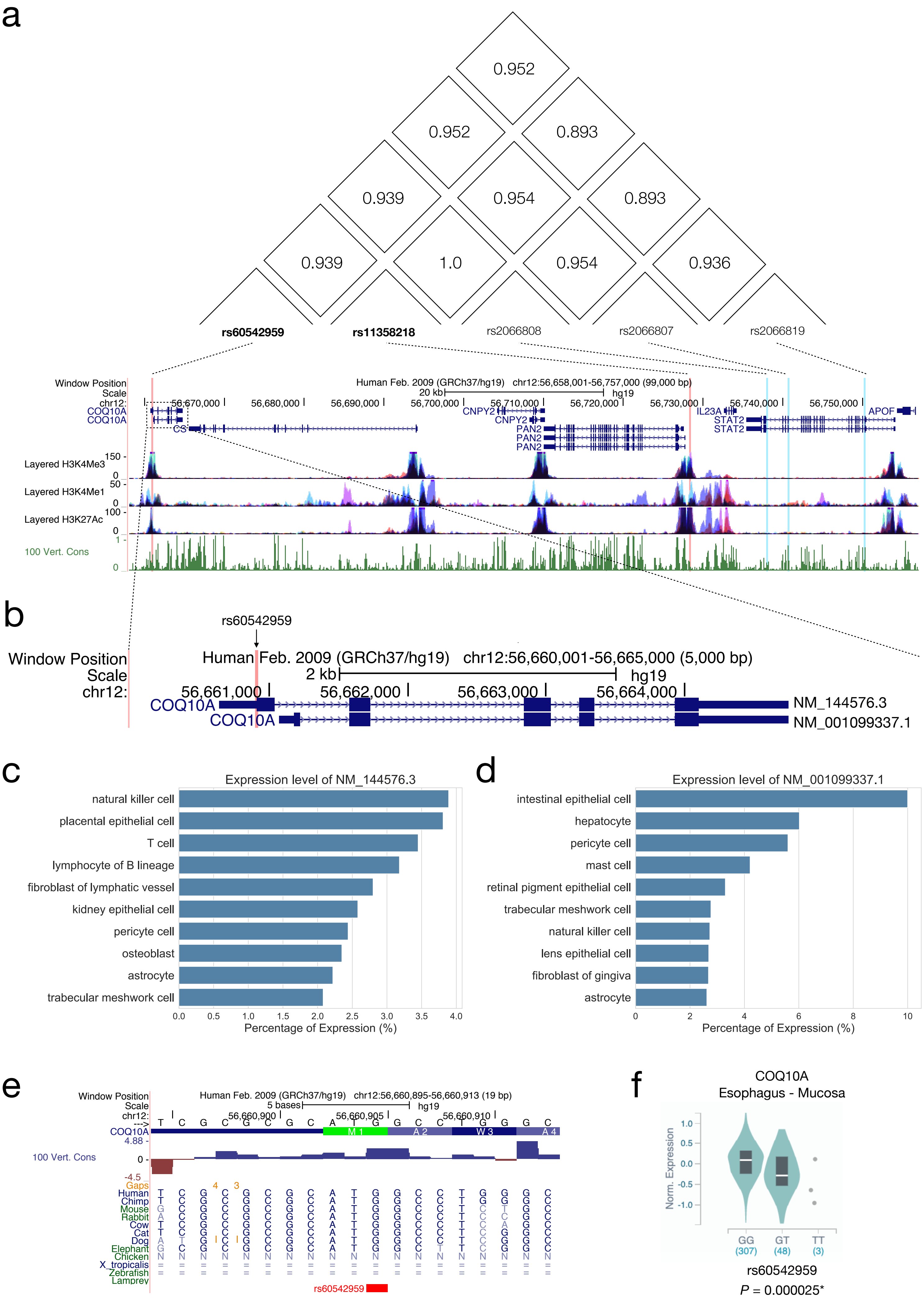
a
0.952
0.952
0.893
0.939
0.954
0.893
0.939
1.0
0.954
0.936
rs60542959
rs11358218
rs2066808
rs2066807
rs2066819
Window Position
Scale
chr12:
Human Feb. 2009 (GRCh37/hg19) chr12:56,658,001-56,757,000 (99,000 bp)
20 kb
hg19
56,670,000
56,680,000
56,690,000
56,700,000
56,710,000
56,720,000
56,730,000
56,740,000
56,750,000
COQ10A
COQ10A
CS
CNPY2
CNPY2
PAN2
PAN2
PAN2
IL23A
STAT2
STAT2
APOF
Layered H3K4Me3
150
0
Layered H3K4Me1
50
0
Layered H3K27Ac
100
0
100 Vert. Cons
1
0
b
rs60542959
Window Position
Scale
chr12:
Human Feb. 2009 (GRCh37/hg19) chr12:56,660,001-56,665,000 (5,000 bp)
2 kb
hg19
56,661,000
56,662,000
56,663,000
56,664,000
COQ10A
COQ10A
NM_144576.3
NM_001099337.1
c
Expression level of NM_144576.3
natural killer cell
placental epithelial cell
T cell
lymphocyte of B lineage
fibroblast of lymphatic vessel
kidney epithelial cell
pericyte cell
osteoblast
astrocyte
trabecular meshwork cell
0.0
0.5
1.0
1.5
2.0
2.5
3.0
3.5
4.0
Percentage of Expression (%)
d
Expression level of NM_001099337.1
intestinal epithelial cell
hepatocyte
pericyte cell
mast cell
retinal pigment epithelial cell
trabecular meshwork cell
natural killer cell
lens epithelial cell
fibroblast of gingiva
astrocyte
0
2
4
6
8
10
Percentage of Expression (%)
e
Window Position
Scale
chr12:
--->
COQ10A
Human Feb. 2009 (GRCh37/hg19) chr12:56,660,895-56,660,913 (19 bp)
5 bases
hg19
56,660,900
56,660,905
56,660,910
T C G C G C G C A T G G C C T G G G C
M 1
A 2
W 3
A 4
4.88 -
100 Vert. Cons
0 -
-4.5
Gaps
4
3
Human
Chimp
Mouse
Rabbit
Cow
Cat
Dog
Elephant
Chicken
X_tropicalis
Zebrafish
Lamprey
rs60542959
f
COQ10A
Esophagus - Mucosa
Norm. Expression
1.0
0.5
0.0
-0.5
-1.0
GG
(307)
GT
(48)
TT
(3)
rs60542959
P = 0.000025*

**Fig. 3** rs60542959 is a deleterious variant that causes a start lost mutation in the *COQ10A* gene.

**a.** The r-squared values between each variant in the European population, of which genotype data originate from Phase 3 (Version 5) of the 1000 Genomes Project, in the 12q13.3 region are shown. The blue and red lines indicate the location of psoriasis GWAS variants and candidate functional variants, respectively. rs60542959 is on the first exon of the *COQ10A* gene, and rs11358218 is located within the *PAN2* gene promoter.

**b.** A magnified view of the *COQ10A* gene locus.

**c, d.** The top 10 cells of CAGE-based expression level of the transcript NM_144576.3 (CAGE_peak_1_at_COQ10A_5end) (c) and NM_001099337.1 (CAGE_peak_3_at_COQ10A_5end) (d). The "Percentage of Expression" for each cell type refers to how much of the total expression (normalized CAGE counts from all cells) the promoter emits for the cell type.

**e.** Genomic sequence alignment among vertebrates of the region around the first codon of the *COQ10A* gene. The location of rs60542959 is indicated by a red box.

f. Comparison of the *COQ10A* expression level among rs60542959 genotypes in the esophagus mucosa. The plot was generated by the GTEx project. The horizontal axis shows genotypes with the number of individuals in parentheses. The asterisk indicates statistical significance.

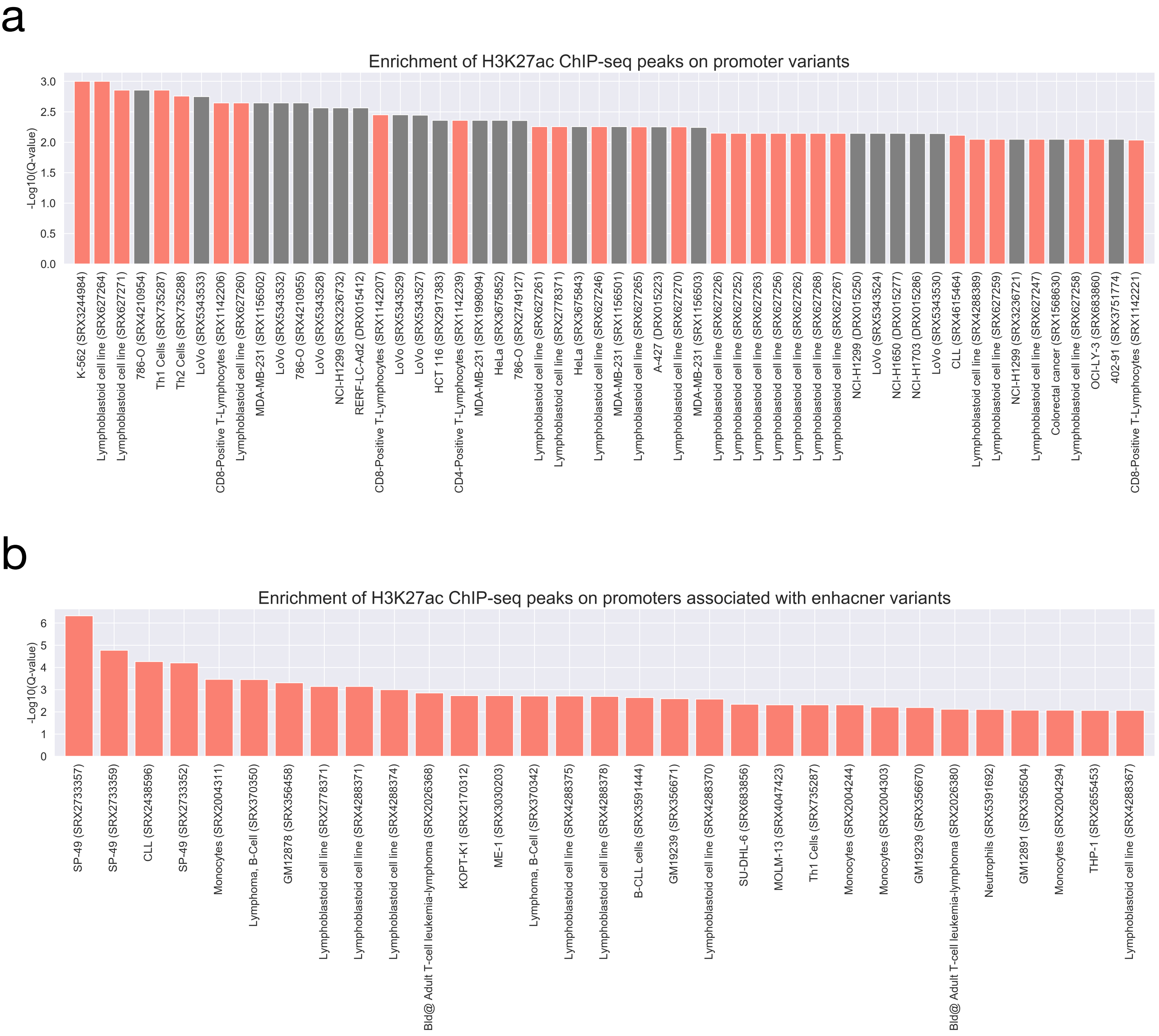


**Fig. 4** H3K27ac ChIP-seq peaks of immune cells are enriched among psoriasis-associated promoter regions. The results of enrichment analyses, of which q-values are more that 0.01, for promoter regions including LD variants (a) and promoter regions associated with enhancers including LD variants (b) are shown in barplots. The horizontal axis represents cell types (the parenthesis indicates experiment IDs). Blood cells are shown in red and other cell types are shown in grey.

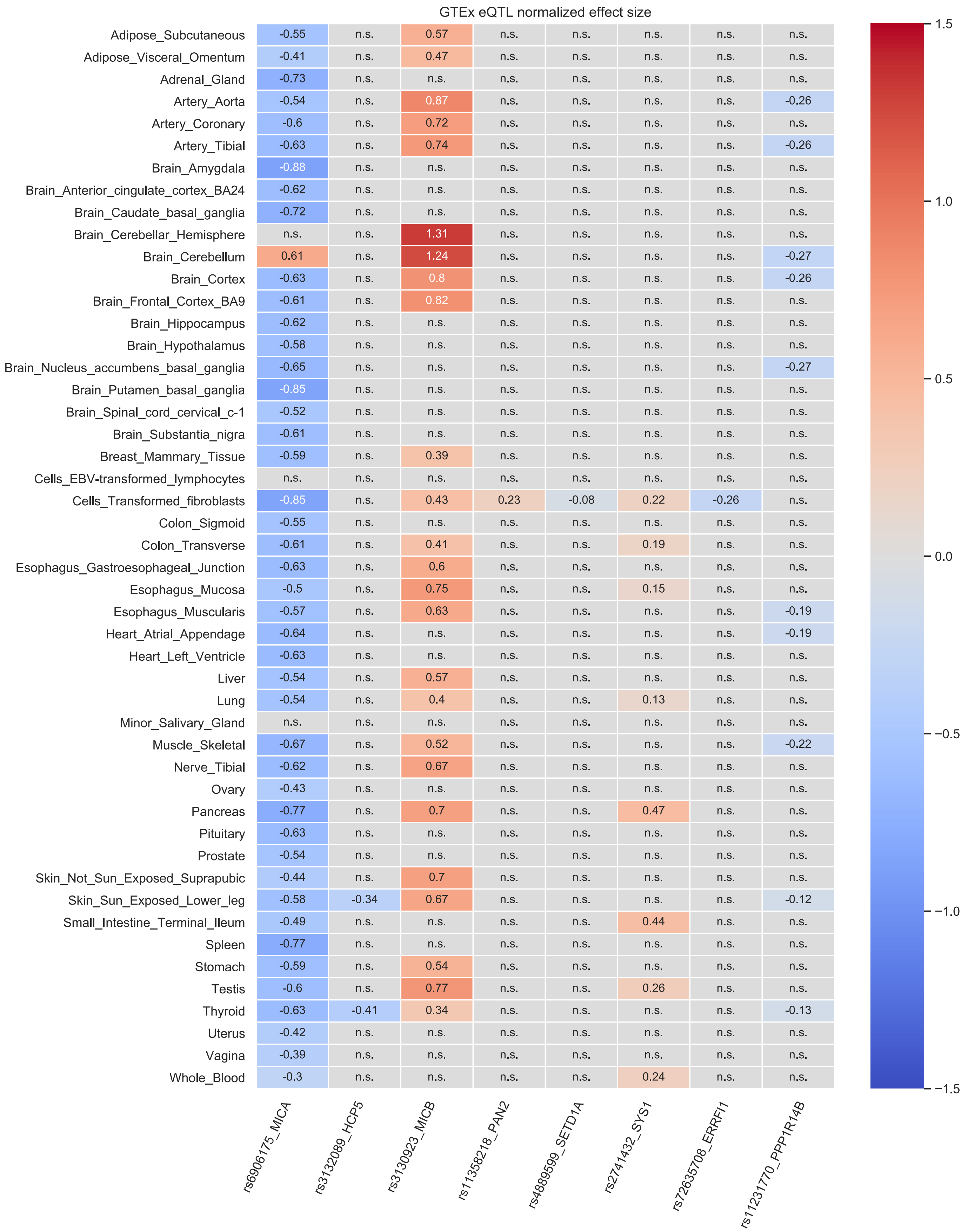


**Fig. 5** A heat map of the normalized effect size of GTEx eQTLs for functional variants in promoters and enhancers.

The vertical axis represents the tissues analyzed, and the horizontal axis represents the functional variants and their target genes. The score for each cell indicates the normalized effect size, which is the effect of the alternative allele for gene expression. Increases in gene expression are shown in red and decreases in gene expression are shown in blue. The “n.s.” in gray cells mean that the effect of allele on gene expression is not statistically significant.

## Do TFs bind to the variants?

| | A549 | GM12878 | H1-hESC | HCT116 | HEK293 | HL-60 | HUVEC cell | HeLa-S3 | HepG2 | IMR-90 | K562 | MCF 10A |
|---|---|---|---|---|---|---|---|---|---|---|---|---|
| REST to rs6906175 (MICA) | No | No | No | No | No | No | No | No | Yes | No | No | No |
| ARNT to rs3132089 (HCP5) | No | No | No | No | No | No | No | No | No | No | Yes | No |
| BHLHE40 to rs3132089 (HCP5) | No | No | No | No | No | No | No | No | Yes | No | Yes | No |
| TCF3 to rs3130923 (MICB) | No | Yes | No | No | No | No | No | No | No | No | No | No |
| SPI1 to rs11358218 (PAN2) | No | No | No | No | No | Yes | No | No | No | No | No | No |
| EGR1 to rs4889599 (SETD1A) | No | Yes | No | No | No | No | No | No | No | No | No | No |
| CEBPB to rs2741432 (SYS1) | Yes | No | No | No | No | No | No | No | Yes | No | No | No |
| ARNT to rs72635708 (ERRFI1) | No | No | No | No | No | No | No | No | No | No | Yes | No |
| BACH1 to rs72635708 (ERRFI1) | No | No | Yes | No | No | No | No | No | No | No | Yes | No |
| FOS to rs72635708 (ERRFI1) | No | No | No | No | No | No | Yes | Yes | No | No | Yes | Yes |
| MAFK to rs72635708 (ERRFI1) | No | No | Yes | No | No | No | No | Yes | Yes | Yes | Yes | No |
| NFE2 to rs72635708 (ERRFI1) | No | No | No | No | No | No | No | No | No | No | Yes | No |
| SP1 to rs11231770 (PPP1R14B) | Yes | No | Yes | Yes | No | No | No | No | No | No | Yes | No |
| SP2 to rs11231770 (PPP1R14B) | No | No | Yes | No | No | No | No | No | No | No | Yes | No |
| ZNF263 to rs11231770 (PPP1R14B) | No | No | No | No | Yes | No | No | No | No | No | No | No |

**Fig. 6** A Table of bindings of transcription factors (TFs) to functional variants in promoters and enhancers in various cells.

The vertical axis represents the TFs, functional variants (the parenthesis indicates the target genes). The horizontal axis represents the cell types. “Yes” indicates the presence of a binding peak of TFs ChIP-seq on the variants in the cells. Conversely, "No" indicates the absence of a binding peak of TFs ChIP-seq

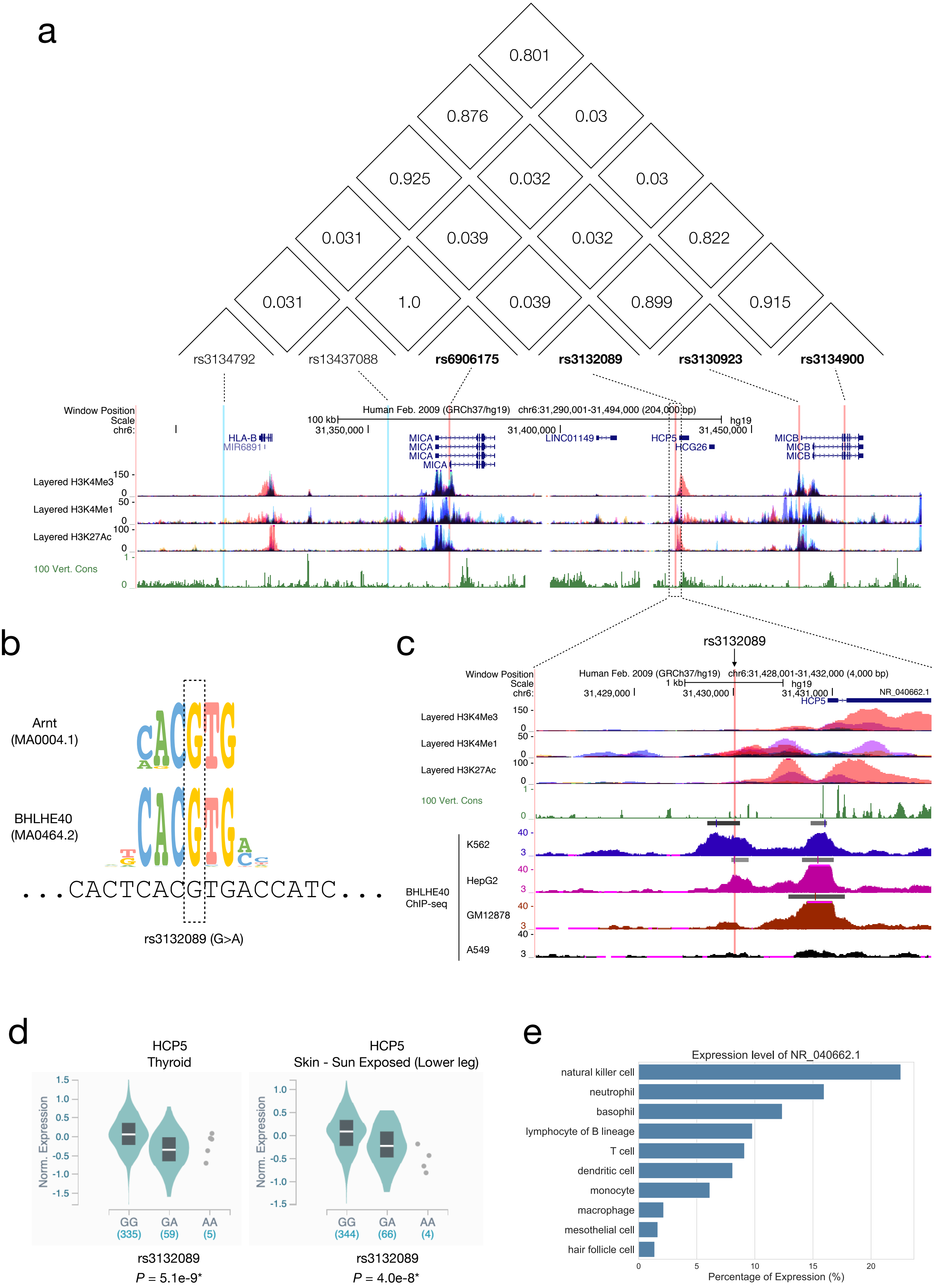
a
0.801
0.876
0.03
0.925
0.032
0.03
0.031
0.039
0.032
0.822
0.031
1.0
0.039
0.899
0.915
rs3134792
rs13437088
rs6906175
rs3132089
rs3130923
rs3134900
Window Position
Scale
chr6:
Human Feb. 2009 (GRCh37/hg19) chr6:31,290,001-31,494,000 (204,000 bp)
100 kb
hg19
31,350,000
31,400,000
31,450,000
HLA-B
MIR6891
MICA
MICA
MICA
MICA
LINC01149
HCP5
HCG26
MICB
MICB
MICB
Layered H3K4Me3
Layered H3K4Me1
Layered H3K27Ac
100 Vert. Cons
b
Arnt
(MA0004.1)
BHLHE40
(MA0464.2)
...CACTCACGTGACCATC...
rs3132089 (G>A)
c
rs3132089
Window Position
Scale
chr6:
Human Feb. 2009 (GRCh37/hg19) chr6:31,428,001-31,432,000 (4,000 bp)
1 kb
hg19
31,429,000
31,430,000
31,431,000
NR_040662.1
HCP5
Layered H3K4Me3
Layered H3K4Me1
Layered H3K27Ac
100 Vert. Cons
K562
HepG2
GM12878
A549
BHLHE40
ChIP-seq
d
HCP5
Thyroid
Norm. Expression
GG
(335)
GA
(59)
AA
(5)
rs3132089
P = 5.1e-9*
HCP5
Skin - Sun Exposed (Lower leg)
Norm. Expression
GG
(344)
GA
(66)
AA
(4)
rs3132089
P = 4.0e-8*
e
Expression level of NR_040662.1
natural killer cell
neutrophil
basophil
lymphocyte of B lineage
T cell
dendritic cell
monocyte
macrophage
mesothelial cell
hair follicle cell
0
5
10
15
20
Percentage of Expression (%)

**Fig. 7** The rs3132089 is a candidate functional variant located within the *HCP5* gene promoter.

**a.** An overview of the HLA region with a genome conservation track (100 vertebrates conservation by PhastCons) and layered ChIP-seq signals of H3K4me3, H3K4me1, and H3K27ac in the UCSC Genome Browser. The vertical light blue lines indicate the location of the psoriasis GWAS variants, and the vertical red lines indicate the location of candidate functional variants. The r-squared values between each variant in the European population, of which genotype data originate from Phase 3 (Version 5) of the 1000 Genomes Project, are shown in blocks.

**b.** The sequence logos based on position weight matrix of binding sites for Arnt (MA0004.1) and BHLHE40 (MA0464.2) are represented with genomic sequences. The location of rs3132089 is indicated by a dotted box.

**c.** A magnified view of the *HCP5* gene (NR_040662.1) promoter with BHLHE40 ChIP-seq signals of K562, HepG2, GM12878, and A549.

**d.** Comparison of the *HCP5* expression level among rs3132089 genotypes in the thyroid and sun exposed skin of lower leg. The plots were generated by the GTEx project. The horizontal axis shows genotypes with the number of individuals in parentheses. The asterisks indicate statistical significance.

**e.** The top 10 cells of CAGE-based expression level of the transcript NR_040662.1 (CAGE_peak_1_at_HCP5_5end). The "Percentage of Expression" for each cell type refers to how much of the total expression (normalized CAGE counts from all cells) the promoter emits for the cell type.

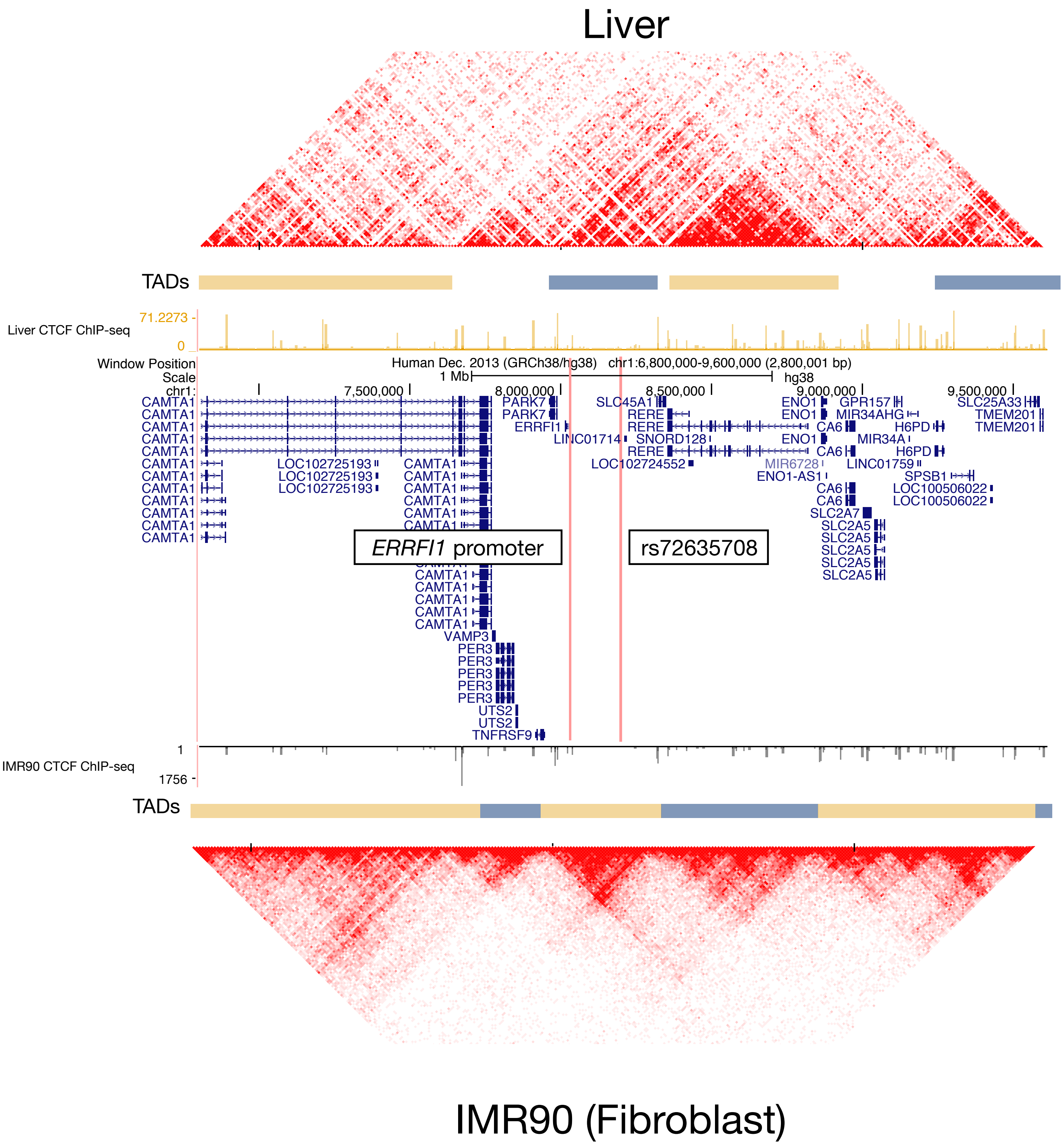


**Fig. 8** Hi-C contact maps for the liver (top) and IMR90 cell line (bottom) in the 1p36 region are shown with RefSeq Curated Genes track and CTCF ChIP-seq signals. The resolution is 10 kb. TADs are indicated by yellow and blue boxes. The red vertical lines indicate the location of the *ERRFI1* promoter and a candidate functional variant, rs72635708, within the enhancer targeting the *ERRFI1* gene.

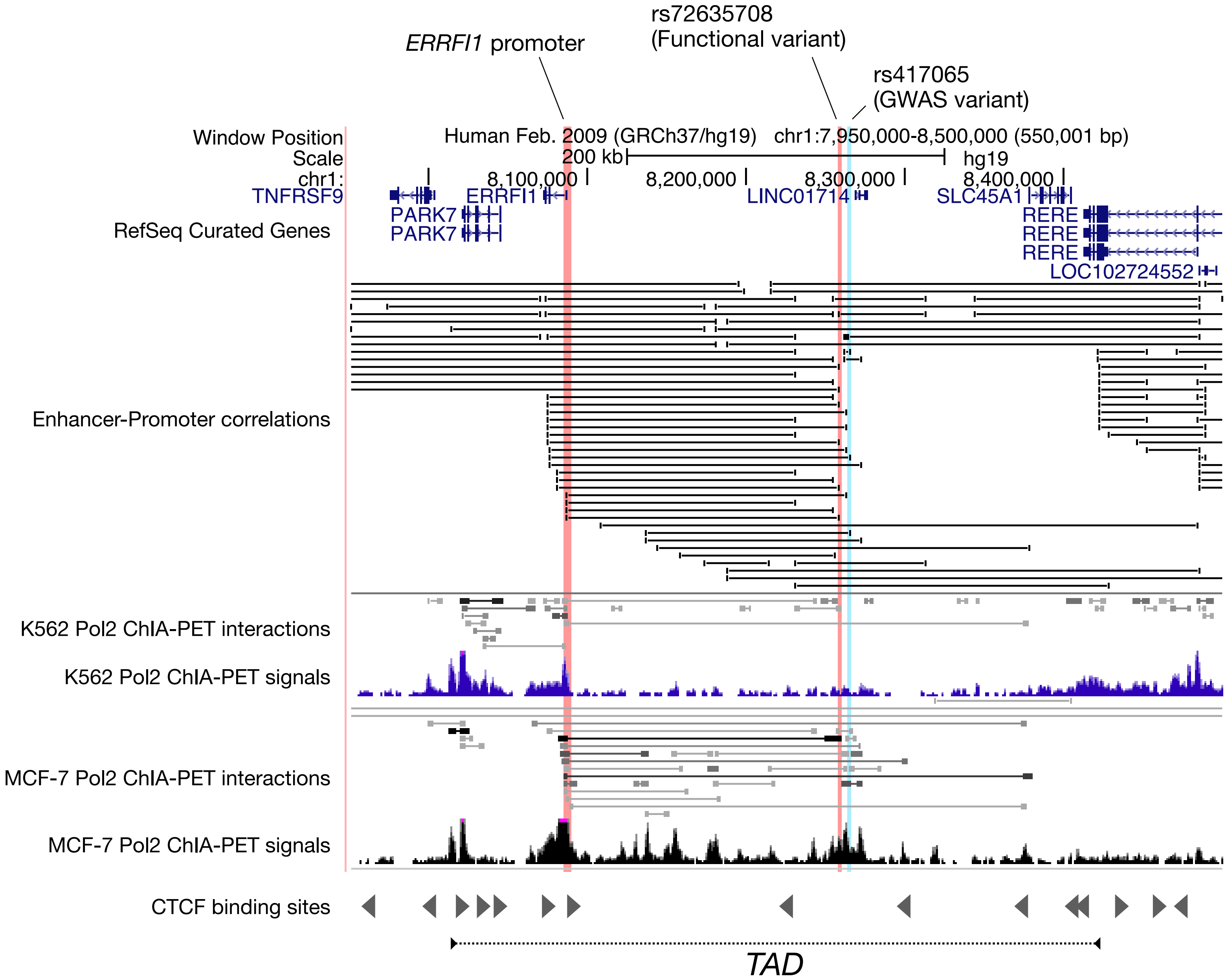


**Fig. 9** An overview of the 1p36.23 region that contains the *ERRFI1* gene and rs72635708. RefSeq Curated Genes track is shown at the top, and enhancer–promoter correlations defined by CAGE datasets are shown by capped lines in the middle panel. Pol II ChIA-PET interactions of K562 and MCF-7 cells are shown with signals in purple and black, respectively. The intensity of interaction lines represents the signal strength. The binding sites of CTCF and its direction are shown at the bottom with a predicted TAD.

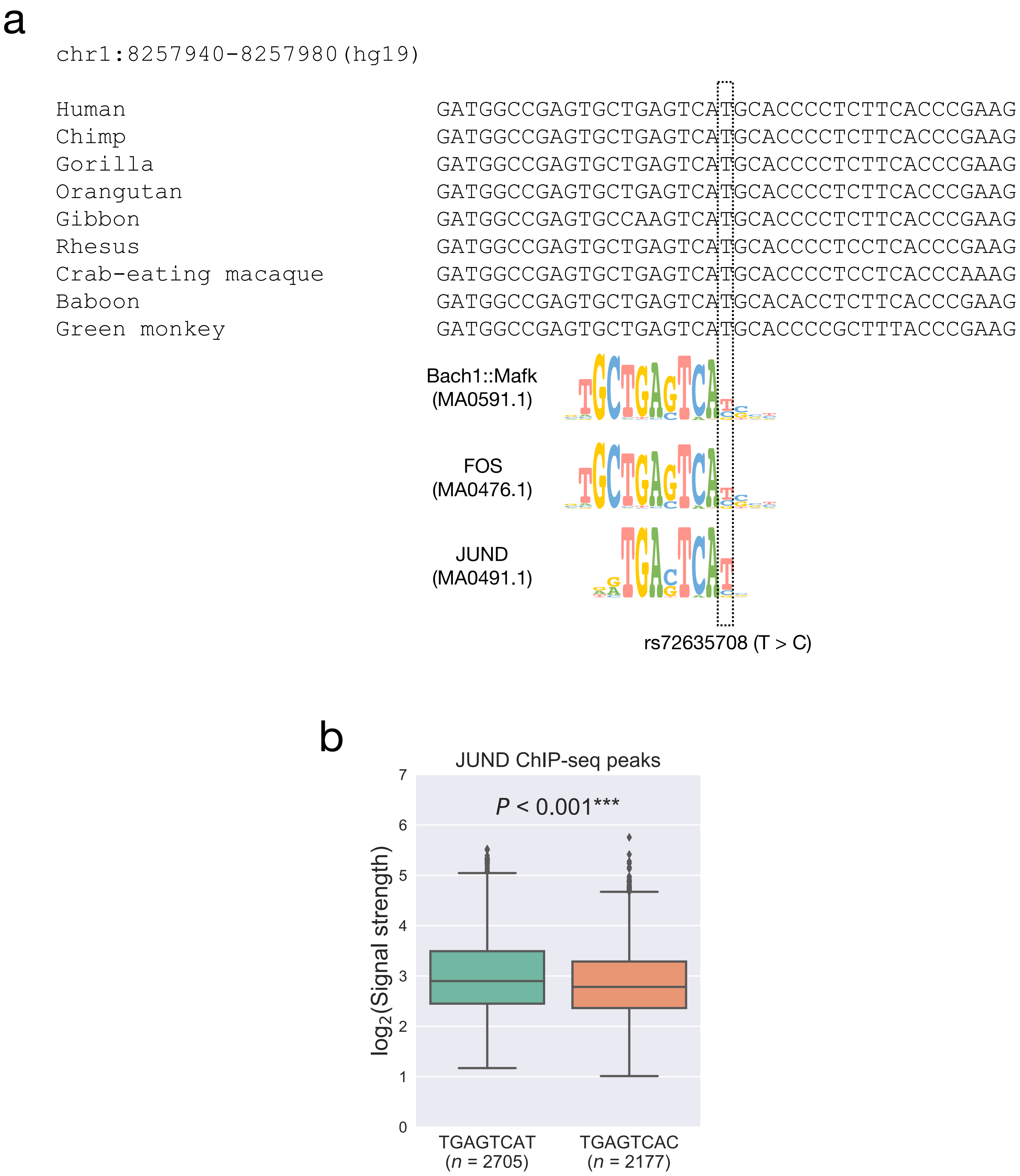


**Fig. 10** The effect of rs72635708 on AP-1 complex binding.

**a.** Genomic sequence alignments among primates of the region near rs72635708, with logos of the binding sites for Bach1::Mafk (MA0591.1), FOS (MA0476.1), and JUND (MA0491.1). The location of rs72635708 is indicated by a dotted box.

**b.** Comparison of JUND binding levels between peaks with the protective motif (TGAGTCAT) and the risk motif (TGAGTCAC) in the liver. The asterisk indicates statistical significance (Mann–Whitney U test).
